# Infantile Neuroaxonal Dystrophy: Case Report and Review of Literature

**DOI:** 10.3390/medicina60081322

**Published:** 2024-08-15

**Authors:** Alian Fatima, Shahd A. Abuhijleh, Abdul Fatah, Mariam M. Mohsin, Subhranshu Sekhar Kar, Rajani Dube, Biji Thomas George, Manjunatha Goud Bellary Kuruba

**Affiliations:** 1 Department of Pediatrics, Saqr Hospital, Ras Al-Khaimah P.O. Box 5450, United Arab Emirates; alianfatima480@gmail.com (A.F.); 212000.shahd@gmail.com (S.A.A.); abdulfatah.5623@gmail.com (A.F.); maryammehwish@gmail.com (M.M.M.); 2 Department of Pediatrics, RAK College of Medical Sciences, RAKMHSU, Ras Al-Khaimah P.O. Box 11172, United Arab Emirates; 3 Department of Obstetrics and Gynecology, RAK College of Medical Sciences, RAKMHSU, Ras Al-Khaimah P.O. Box 11172, United Arab Emirates; rajani.dube@rakmhsu.ac.ae; 4 Department of General Surgery, RAK College of Medical Sciences, RAKMHSU, Ras Al-Khaimah P.O. Box 11172, United Arab Emirates; biji@rakmhsu.ac.ae; 5 Department of Biochemistry, RAK College of Medical Sciences, RAKMHSU, Ras Al-Khaimah P.O. Box 11172, United Arab Emirates; manjunatha@rakmhsu.ac.ae

**Keywords:** neurodegenerative disorder, infantile neuroaxonal dystrophy, developmental regression, global developmental delay, genetic counseling

## Abstract

Infantile neuroaxonal dystrophy (INAD) is a rare neurodegenerative disorder affecting 1:1,000,000 children. It results from pathogenic variants in the PLA2G6 gene located on chromosome 22q13.1. The onset of symptoms usually occurs between 6 and 18 months, causing developmental regression leading to debilitating symptoms such as muscle weakness, dementia, and loss of basic skills. Eventually, it progresses to life-threatening symptoms, including breathing difficulties, which limit the life expectancy to 5–10 years. While potential genetic therapies for treatment are being developed, they are yet to be approved for use, and management remains essentially supportive. This case report is about a nine-year-old Pakistani girl with INAD. She presented with recurrent chest infections, developmental regression, loss of speech, paralysis, hypertension, and eventually breathing difficulties. Brain magnetic resonance imaging and genetic testing confirmed the diagnosis. This case posed diagnostic challenges in view of its overlapping clinical presentation. Through this report, we aim to raise awareness about this condition among practitioners, outline the importance of genetic counseling in susceptible couples, and suggest potential areas of further research.

## 1. Introduction

Infantile neuroaxonal dystrophy (INAD, NBIA2A; MIM#256600) is a rapidly progressive neurodegenerative disorder. It was first described by Seitelberger in 1952, granting it the name Seitelberger disease. INAD is very rare, estimated to affect 1:1,000,000 children worldwide [1]. It is characterized by psychomotor regression presenting in early infancy, with symptoms reflecting the involvement of peripheral nerves, the central nervous system, and the autonomic nervous system.

INAD results from pathogenic variants in the *PLA2G6* gene located on chromosome 22q13.1 and is inherited as autosomal recessive [2]. It is a subtype of PLA2G6-associated neurodegeneration (PLAN). Other conditions in this disease group include Karak syndrome, juvenile-adult-onset dystonia-parkinsonism (PARK14; MIM# 612953), and atypical neuroaxonal dystrophy (NAD, NBIA2B; MIM#610217) [1,2]. As a subtype of PLAN, the pathophysiology of INAD has been described to be related to pathogenic variations in the *PLA2G6* gene, which encodes calcium-independent phospholipase A2. This enzyme hydrolyses glycerophospholipids, contributing an essential step in axonal and synaptic phospholipid membrane remodeling. Pathogenic variations lead to the inability to repair oxidative damage to membranes [2].

The natural progression of this disease has been described by multiple studies, with the onset of symptoms between 6 months and 3 years with an average of 15 months [3,4]. Another describes a range of 12 to 22 months [4]. The initial presentation of the disease is usually a loss of balance and developmental delay [3]. Developmental regression initially affects speech and gross motor functions, followed later by the affection of fine motor and bulbar functions. This usually manifests as truncal hypotonia, spastic quadriparesis, progressive cognitive decline, loss of vocalization, and bulbar dysfunctions. Seizures may be present early or late in the disease course, or not at all. Atypical INAD presents with similar but more subtle clinical features compared to INAD, such as slower progression [5]. The disease has a poor prognosis. Patients most often succumb to respiratory function decline secondary to bulbar affection, with an average age at death of 9.9 years (range of 6.5–14 years) [3,4]. There are no specific curative treatments available for patients with INAD. They are treated conservatively with analgesics and physiotherapy with vigilance for complications. However, newer therapeutic options are currently being explored.

We report a rare case of INAD in a nine-year-old girl presenting with overlapping features, posing significant diagnostic challenges. We aim to spread awareness regarding this condition among practitioners, facilitate early identification, and outline the importance of appropriate genetic counseling.

## 2. Case Presentation

A nine-year-old Pakistani girl presented with recurrent pneumonia, with a prior known history of global developmental delay and psychomotor regression since she was 15 months old. The child was born to parents with first-degree consanguineous marriage. The pregnancy was planned with an uncomplicated antenatal period, vaginal delivery at term, and a birth weight of 2.7 kg. The immediate postnatal course was uneventful without neonatal intensive care unit (ICU) admissions. She was breastfed till the age of one, and received all age-appropriate vaccinations.

She was diagnosed with developmental dysplasia of the hip (DDH) and bilateral dislocation of the hips by an X-ray and ultrasonography during early infancy and was managed nonsurgically with a Pavlik harness. Her developmental milestones in gross motor, fine motor, social, and language skills were normal until 15 months of age. Then, she presented with tip-toe walking and gross motor delay followed by the progressive loss of various developmental milestones in other domains. Her paternal cousin is suspected to be suffering from a similar illness due to developmental delays, without any confirmation of diagnosis so far due to the limited resources. All her other siblings have normal growth and development.

Despite her normal growth, concerns arose when she started tip-toe walking at 15 months of age, with complaints of aching in her legs, slowly causing her to avoid walking around. At the age of two, her symptoms rapidly deteriorated, causing her to lose her ability to walk steadily and sit upright, ultimately resorting to crawling as her only means of movement. Her cognitive capacity to perceive and process motor tasks was diminishing, resulting in the loss of her pincer and palmar grasp, as well as an inability to reach for objects. However, her hearing and visual senses remained partially intact, enabling her to react to loud sounds and perceive bright lights. She struggled to respond to conversations, and her social interactions were often negative. Moreover, she progressively showed signs of intellectual disability. Beyond the usual symptoms, she experienced prolonged seizure attacks, recurrent pneumonia, and resistant hypertension. In addition to these, she had frequent medical encounters for multiple reasons, including diaper rashes, recurrent bed sores, skin abrasions, and chronic constipation. There was a history of multiple admissions into different hospitals and the ICU, with problems including but not limited to recurrent seizures, hypertension, chronic gastroesophageal reflux disease, recurrent urinary and respiratory infections, obstructive sleep apnea, and respiratory distress throughout her childhood.

On examination, she had elevated blood pressure. A neurological examination revealed spastic quadriplegia, augmented tendon reflexes, and bilateral positive Babinski sign signifying symmetric pyramidal tract involvement. Her lower limb sensations were completely absent, but the reaction to painful stimuli was present in both the upper limbs. Other systemic examinations were unremarkable. Her investigations included complete blood count (CBC), urinalysis, electrolytes, renal function tests (RFT), liver function tests, C-reactive protein (CRP), and procalcitonin, with results within normal limits for her age. An EEG revealed nonspecific findings. Additional investigations, such as arterial blood gas (ABG) analysis, urine cultures, and cerebrospinal fluid (CSF) analysis from a lumbar puncture (LP), showed no significant abnormalities, ruling out systemic infections or metabolic derangements. Further investigations such as a nerve biopsy and an EMG were not performed.

**Figure 1:**
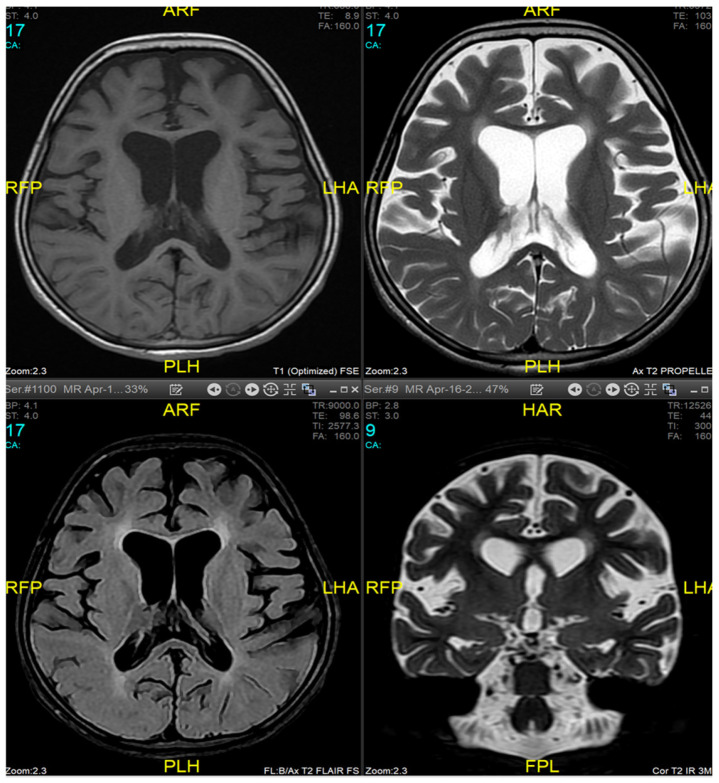
Brain MRI at 9 years of age (16 April 2024). Generalized atrophic changes of the brain, characterized by supra and infratentorial ventricular dilatation, prominent cortical sulci, and extra-axial CSF spaces such as Sylvian fissures and basal cisterns. The diffuse deep periventricular white matter showed high signal intensities on T2/FLAIR (fluid-attenuated inversion recovery) sequences. There was global thinning of the corpus callosum.

**Figure 2:**
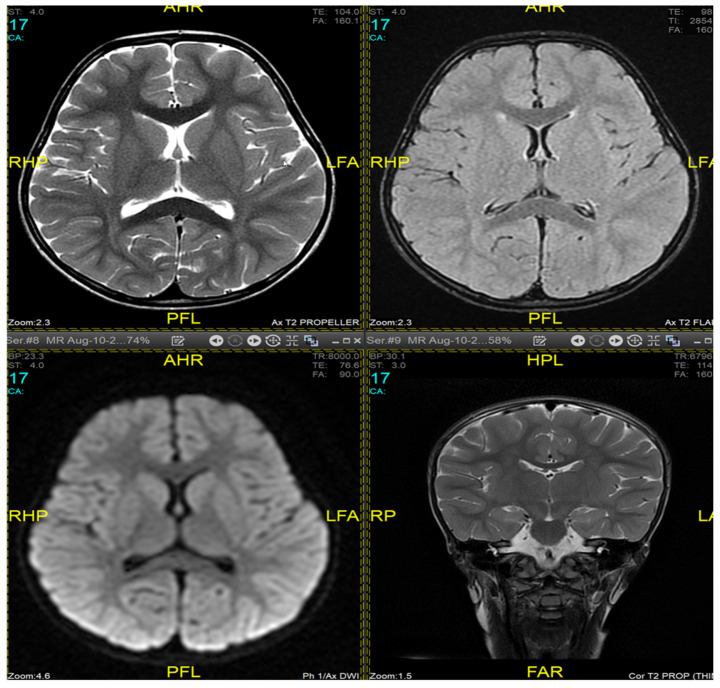
Brain MRI at 2.5 years old (10 August 2017). No evidence of atrophic brain changes or iron deposition.

Magnetic resonance imaging (MRI) of the brain and genetic analysis were performed to support the diagnosis. Her MRI showed newly developed generalized atrophic changes of the brain, characterized by supra and infratentorial ventricular dilatation, prominent cortical sulci, and extra-axial CSF spaces such as Sylvian fissures and basal cisterns. The diffuse deep periventricular white matter showed high signal intensities on T2/FLAIR (fluid-attenuated inversion recovery) sequences. Additionally, there was global thinning of the corpus callosum [Figure 1]. This was in contrast to her prior brain MRI at 2.5 years old, which showed no evidence of atrophic brain changes or iron deposition [Figure 2].

Her molecular genetic analysis of whole exome sequencing (WES) at 3.5 years old identified the homozygous variant c.68G>A p.(Arg23Gln) chr22:38565366 on the *PLA2G6* gene, which is known to be the pathogenic variant of INAD [6]. Parallel analysis of parental WES revealed that both parents are heterozygous carriers.

She received supportive and targeted treatment for her problems, involving a multidisciplinary team (MDT) aimed to prevent complications resulting from INAD. The received treatment included surgical interventions like PEG tube insertion, adenoidectomy, the antiseizure medication Levetiracetam to control her seizures, topical/systemic antibiotics with wound care for bed sores, Amlodipine for hypertension, and Pantoprazole for her gastrointestinal complaints. She then underwent tracheostomy to address her respiratory complications.

## 3. Discussion

Confusing clinical findings, along with the social and environmental factors, resulted in a delayed diagnosis in our case [7]. Neuroaxonal dystrophies are a group of neurodegenerative diseases with an autosomal recessive inheritance pattern. Intrauterine infections can result in structural and functional abnormalities of the central nervous system [8,9]. Events in early neonatal life have not been found to affect the onset or clinical picture of INAD; however, postnatal complications were found to occur more frequently in INAD patients when compared to the general population [3]. Our patient did not have any complications in the antenatal, intranatal, or postnatal period, and presented for the first time at 15 months of age. This is in accordance with another descriptive study involving 28 cases from different countries, where the average age of presentation was reported to be 15 months (range of 6 months–3 years) [3]. The natural history of the disease was reported in a study where speech impairment and the loss of gross motor milestones were the earliest signs of the disease [2,3]. Nystagmus, seizure, gastrointestinal symptoms, skeletal deformities, and strabismus were the other reported symptoms [2,3]. Similarly, features of autonomic nervous system involvement like constipation, urinary retention or incontinence, reduced tear production, and temperature dysregulation were reported in patients of INAD by another study [2]. In our patient, speech and hearing impairment presented later. Although our patient showed signs of motor impairment and seizures, there were no facial dyskinesia, tongue fasciculations, microcephaly, ataxia, tremors, strabismus, and nystagmus. Our patient exhibited hypertension, unlike other case reports. The differential diagnosis in children with similar features of severe psychomotor disability, seizures, optic atrophy, blindness, and marked long-tract signs, at the age of around three can be a lysosomal storage disorder like Schindler disease type I, which is caused by mutations in a-N-acetylgalactosaminidase (NAGA) [10,11,12]. The MRI may also show diffuse white matter changes and demyelination. This condition is diagnosed by the estimation of oligosaccharide levels in urine and assays of NAGA activity in leukocytes or fibroblasts [10,12,13,14,15]. Urinary oligosaccharide levels were normal in our patient.

In 1979, a set of diagnostic criteria for INAD were defined based on an analysis of clinicopathological findings in 50 patients summarized as follows: (i) onset before 3 years, (ii) the progressive course of diffuse central nervous system disorder with psychomotor deterioration and increasing neurological involvement comprising symmetrical pyramidal tract signs and marked hypotonia, and finally (iii) the presence of axonal spheroids on biopsy of central or peripheral nervous system tissue [7]. The role of MRI was not initially considered for this analysis. With the inclusion of imaging results, MRI features were considered by another study [16]. Finally, with the advancement of molecular diagnostic techniques, skin biopsies are no longer used regularly and WES is currently playing a vital role in diagnosing patients with INAD by identifying pathological variants of the *PLA2G6* gene [5]. It is to be noted that various mechanisms for genetic changes in the gene exist and all of them may not have been explored yet. The mutations reported in the literature are homozygous mutations (MIM *603604) involving the gene-encoding phospholipase A2 (PLA2) group VI (cytosolic, calcium-independent [Entrez Protein accession number NP_003551.2]) [17], in the position C.2370 T>G (p. Y790X) and C. T2208G in the *PLA2G6* gene [7]. Compound heterozygous mutations c.1798C>T; p.R546W (rs368008077) + c.2357C>T; and p.R741W (rs530348521) were also reported in another case [5]. Furthermore, a study of 22 Indian families with INAD, ANAD, and DPC failed to detect any mutation in the *PLA2G6* gene-coding region in 45% of cases [18].

MRI findings are variable depending on the disease stage at which imaging was performed [1,3,10,12]. Imaging may be completely normal in some patients, as was the case in our patient during the first presentation, making it insufficient to rule out INAD based on radiological findings only and necessitating follow-up imaging later. Typical findings described include cerebral atrophy, white matter changes, and iron deposits in the globus pallidus, substantia nigra, and dentate nucleus [19,20]. Due to the presence of iron deposits, INAD can be classified as a subtype of neurodegeneration with brain iron accumulation diseases (NBIA). The alteration of mitochondrial functions may affect mitochondrial iron homeostasis, leading to neurodegeneration [21]. Defects in transferrin receptor recycling were established to be a common anomaly in fibroblasts from different subtypes of NBIA patients [22], suggesting impaired iron incorporation as a shared mechanism responsible for iron overload in these pathologies. However, iron deposits are not specific to INAD [23]. Our patient exhibited typical MRI features in a follow-up study but did not have any evidence of iron accumulation in the brain.

Management of INAD remains largely supportive, with no cure available yet. Possible curative therapies explored for a rare disease like INAD involving a defective enzyme are enzyme replacement, gene replacement, and gene correction [24]. There are specific procedural challenges to enzyme replacement in the brain, to reach the mitochondria, and in the efficacy of the treatment. A recent study reported the successful use of Adeno-associated virus-based gene therapy in patient-derived neural progenitor cells in flies and mice in slowing the progress of INAD [25]. However, human studies are underway, and results are expected. With the advancement of whole genome sequencing, newer genes are likely to be identified as a cause of INAD and can be targeted for gene therapy. For parents at risk of having an INAD-affected child, it is important to provide preconception counseling and parental carrier testing to accurately predict recurrence risk [26]. Subsequently, offering parents preimplantation genetic testing [27] or prenatal testing [28,29], which have been previously performed successfully, is of importance. With the development of medical technology capable of detecting carriers and easily performing genetic testing, at-risk couples may be identified and appropriately counseled on the disease before conception [29]. It is necessary to report observations and disease progress of rare diseases for the development of novel therapeutic and investigative approaches in the future, and ultimately improve patient outcomes. We urge medical practitioners to consider conditions such as INAD as a possible cause of developmental regression in presenting patients, such as described in our case.

## 4. Conclusions

INAD is a very rare progressive neurodegenerative disorder presenting in early childhood, with reduced life expectancy. Awareness about the disease will help in the early diagnosis of this condition, prevention of complications, and appropriate parental counseling. There is no curative treatment available for INAD yet. There is a need for research to formulate targeted treatment and care plans for patients with this disease. Future research should also be targeted at identifying all genes responsible for INAD by whole genome sequencing and the success of human trials for specific gene therapy.

## Data Availability

No new data were created or analyzed in this study. Data sharing is not applicable to this article.
